# Letter to the Editor Regarding the Article by Karami et al. on the Surveillance of Endemic Coronaviruses During the COVID‐19 Pandemic in Iran, 2021–2022

**DOI:** 10.1111/irv.70001

**Published:** 2024-08-20

**Authors:** Eleni Rousogianni, Garyfallia Perlepe, Stylianos Boutlas, Dimitrios Papagiannis, Konstantinos I. Gourgoulianis

**Affiliations:** ^1^ Department of Respiratory Medicine, Faculty of Medicine, School of Health Sciences University of Thessaly Larissa Greece; ^2^ Emergency Department University Hospital of Larissa Greece; ^3^ Public Health & Vaccines Laboratory, Department of Nursing, School of Health Sciences University of Thessaly Larissa Greece

**Keywords:** surveillance, coronaviruses, seasonality

Sir,

We read with great interest the article by Karami et al. [1] that describes the prevalence of respiratory infections caused by endemic human coronaviruses (eHCoVs) during the COVID‐19 pandemic in Iran, indicating their low prevalence.

The COVID‐19 pandemic has sparked a renewed interest in eHCoVs, which account for an estimated 5%–10% of acute respiratory infections (ARI) in temperate climates [2]. These endemic human coronaviruses are widespread globally and generally result in mild to moderate infections of the upper respiratory tract. However, systematic surveillance data on the epidemiology of endemic coronaviruses NL63, HKU1, OC43, and 229E are currently lacking due to their underappreciated clinical and epidemiological impact. In pre‐pandemic years, respiratory virus detections followed a stable seasonal pattern. Many studies, including the one discussed here, showed the low circulation of seasonal respiratory viruses, especially endemic coronaviruses, during the COVID‐19 pandemic [1, 2]. This was followed by a marked increase after the first and second lockdowns [3]. Compared to 2019, respiratory virus numbers increased by 23% in 2020, 100% in 2021, and 270% in 2022 [3].

This reduction in virus circulation is mainly attributed to nonpharmaceutical interventions (NPIs) like travel restrictions, temporary lockdowns, school closures, mask‐wearing, and improved hygiene practices. These measures likely played an important role in decreasing coronavirus transmission [2]. However, evaluating the impact of NPIs is complex and influenced by factors such as pathogen characteristics, demographics, and timing and location of NPIs. Competition between SARS‐CoV‐2 and other viruses may also affect epidemiology [4]. Additionally, prolonged periods of reduced exposure to common pathogens can lead to a temporary decrease in population immunity. With reduced exposure during the pandemic, people's immune systems might not be as prepared to fend off these endemic viruses once they re‐emerge [5]. The pandemic disrupted the usual circulation patterns of many viruses, allowing some viruses suppressed during the pandemic to find new opportunities to spread once normal activities resumed.

After the low circulation of eHCoVs during the COVID‐19 pandemic, a seasonal increase in respiratory pathogens is expected. As it is mentioned in the Karami et al. article, surveillance research is essential to monitor eHCoV distribution patterns and identify changes in the epidemiology of these viruses. This is essential for developing strategies to timely control the future outbreaks of eHCoVs throughout the nation [1]. In this direction, we examined all adults with acute respiratory infection symptoms presenting to the Emergency and Respiratory Medicine Departments of the University Hospital of Larissa (UHL), Greece, between November 2023 and May 2024. We collected nasopharyngeal swabs and tested for 16 respiratory viruses using multiplex real‐time polymerase chain reaction (PCR) assays. This approach provided insight into the prevalence and seasonal patterns of endemic coronavirus infections during Greece's 2023–2024 season. We then compared these findings with those related to SARS‐CoV‐2.

A total of 2,651 specimens were collected. Out of them, there were 259 (9.77%) coronavirus‐positive specimens and 1,226 (46.25%) specimens positive for 10 other seasonal respiratory viruses. One hundred five (3.96%) samples were positive for SARS‐CoV‐2. Endemic coronaviruses of all four types were detected in 154 (5.81%) of the samples (Figure 1), representing a slight increase compared to the 4.75% reported in the Karami et al. study. A *t*‐test was conducted, and this difference was not found statistically significant (*p*‐value > 0.05). Among endemic coronaviruses OC43 was the most prevalent, with 62 (2.34%) positive specimens. HKU1 and NL63 were detected in lower numbers with 39 (1.47%) and 36 (1.36%) positive specimens, respectively. Only 17 (0.64%) 229E positive specimens were detected.

**FIGURE 1:**
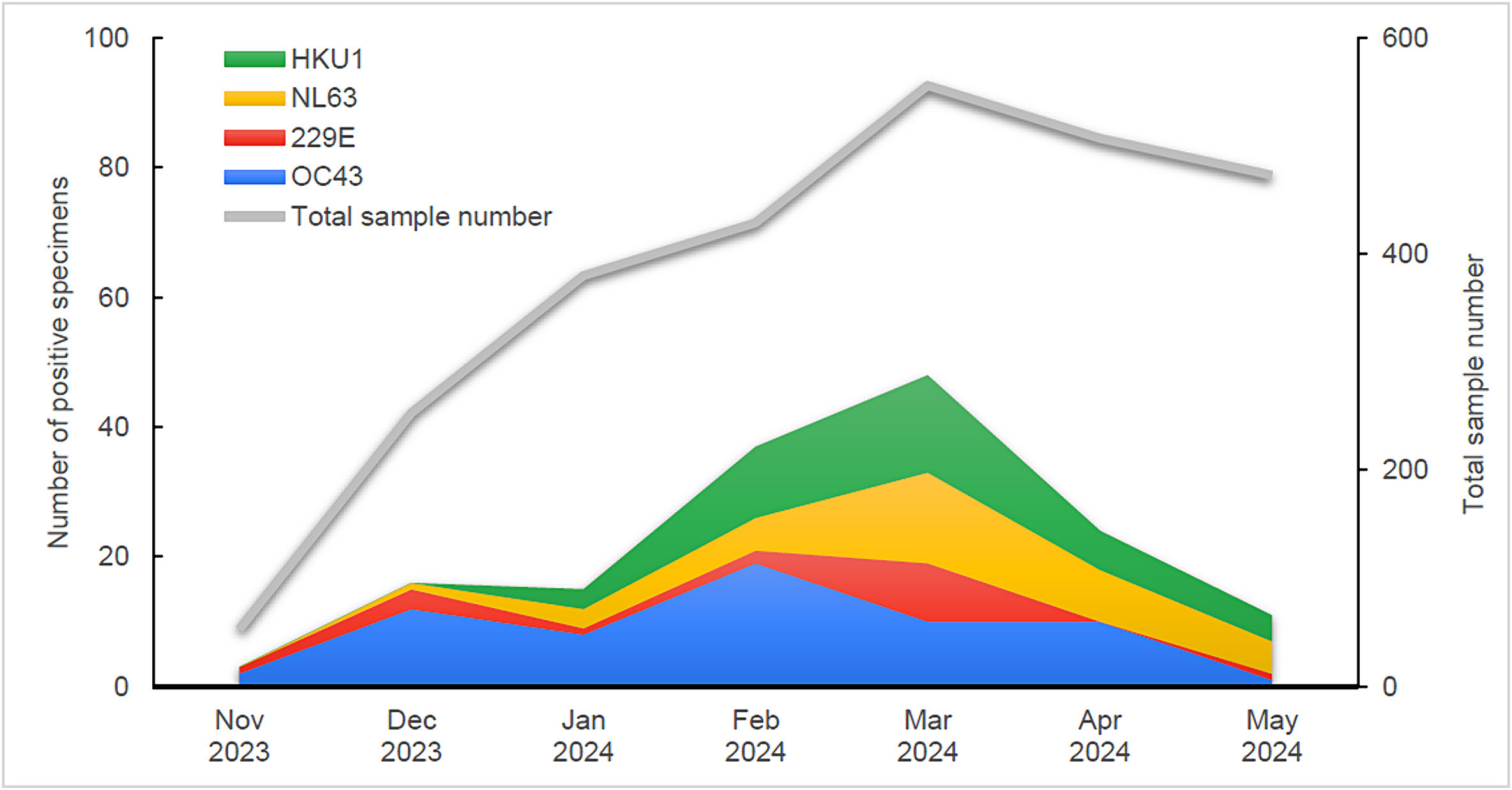
Number of positive specimens for the endemic coronavirus types via multiplex real‐time PCR assays in patients with symptoms of respiratory infection presenting in the Emergency Department and Respiratory Medicine Department of the University Hospital of Larissa, Greece, for 2023–2024, per calendar month.

Overall endemic coronavirus infections peaked in March 2024, when almost 1.8% (48/2651) of all samples contained these viruses. Karami et al. reported a slightly earlier seasonality, with a peak during December 2021 to February 2022. This may be attributable to the COVID‐19 pandemic's impact on the seasonality of respiratory viruses, which now appear to be reverting to their pre‐pandemic patterns. OC43 was predominant, representing 23.94% of total coronavirus infections (62/259), detected throughout the period studied, possibly peaking in February 2024. The other three endemic coronavirus types peaked simultaneously in March 2024. 229E was detected only sporadically in winter and early spring.

In conclusion, our results indicate a slight rise in endemic coronavirus prevalence following the COVID‐19 pandemic, with a peak in early spring. This can be attributed to the relaxation of NPIs that were in place during the pandemic, as well as a decline in immunity due to reduced exposure to pathogens during that time. OC43 was the most prevalent among endemic coronaviruses, whereas the other three coronaviruses were all detected at lower percentages.

## Author Contributions

**Eleni Rousogianni:** investigation, formal analysis, writing – original draft, writing – review and editing. **Garyfallia Perlepe:** writing – review and editing, writing – original draft. **Stylianos Boutlas:** investigation. **Dimitrios Papagiannis:** conceptualization, supervision, project administration. **Konstantinos Gourgoulianis I:** conceptualization, project administration, supervision.
